# Restless Legs Syndrome as a Comorbidity in Rheumatoid Arthritis

**DOI:** 10.1155/2013/352782

**Published:** 2013-06-12

**Authors:** John A. Gjevre, Regina M. Taylor Gjevre

**Affiliations:** Department of Medicine, University of Saskatchewan, Saskatoon, SK, Canada S7N0W8

## Abstract

Rheumatoid arthritis (RA) is a multisystem disease with a complex immunologic pathophysiology. Likewise, sleep disorders can involve a complicated interplay between the neurologic pathways, immune system, and respiratory system. Recent studies have shown an elevated prevalence of sleep abnormalities in connective tissue disorders compared to the general population. Restless legs syndrome (RLS) may be present in up to 30% of RA patients. These findings may be related to cytokine release and other immunomodulatory responses. TNF-**α** levels relate to sleep physiology and anti-TNF-**α** therapy may improve sleep patterns. Most of the patients with this disorder can distinguish their RLS sensations from their arthritic symptoms. RLS is a common comorbidity seen with RA, and prompt recognition and treatment can improve patient quality of life.

## 1. Introduction

Restless legs syndrome (RLS), also called Willis-Ekbom's disease, is a common clinical syndrome which consists of an uncomfortable sensation in one's legs and an irresistible desire to move them usually occurring in the evening. This disorder has been underrecognized and misunderstood by many health care providers as well as the general public. While the original description of RLS was first reported by Dr. Karl Ekbom in 1945, this entity was mainly ignored by clinicians and researchers until the late 1980s [1]. Due to the lack of clear criteria, the International Restless Legs Syndrome Study Group (IRLSSG) was formed and developed clinical criteria in 1995 [2]. Subsequently, the IRLSSG has released revised guidelines to help in the clinical diagnosis and epidemiologic research of RLS [3]. This has helped to both significantly raise awareness of this disorder as well as to further basic scientific and clinical research into RLS.

## 2. RLS Diagnosis and Background

The Willis-Ekbom (RLS) Foundation describes this disease as a disruptive neurologic disorder that seriously affects 2-3% of the population and may affect up to 10% of the U.S. population [4]. Patients will describe an uncomfortable itching or “creepy-crawling” sensation on the legs in the evenings, and this may impact negatively on sleep and quality of life. The 2003 IRLSSG criteria originally listed four essential criteria to clinically diagnose RLS with a 2011 revision adding a fifth criteria.

*2011 Revised IRLSSG Diagnostic Criteria*

1. An urge to move the legs usually but not always accompanied by or felt to be caused by uncomfortable or unpleasant sensations in the legs.
2. The urge to move the legs and any accompanying unpleasant sensations begin or worsen during periods of rest or inactivity such as lying down or sitting.
3. The urge to move the legs and any accompanying unpleasant sensations are partially or totally relieved by movement, such as walking or stretching, at least as long as the activity continues.
4. The urge to move the legs and any accompanying unpleasant sensations during rest or inactivity only occur or are worse in the evening or night than during the day.
5. The occurrence of the previous features is not solely accounted for as symptoms primary to another medical or behavioral condition (e.g., myalgia, venous stasis, leg edema, arthritis, leg cramps, positional discomfort, and habitual foot tapping).

The diagnosis is based on the clinical history using these IRLSSG criteria. There are no simple tests by which the diagnosis can be made although many patients may have iron deficiency with low ferritin levels. Overnight polysomnography (PSG) in a sleep disorders laboratory may be useful to assess periodic limb movements of sleep (PLMS). However, a PSG is not required for the diagnosis of RLS. PLMS are repetitive stereotypical limb movements during sleep associated with electromyographic activity and are a related but separate disease process. Most patients (80%) with RLS will have PLMS on the PSG study, but it is not diagnostic as only one-third of patients with PLMS have clinical RLS symptoms [5]. Unfortunately, while the specificity of the four IRLSSG diagnostic criteria is good at 84%, it can be difficult to exclude mimics (e.g., leg cramps) which may confound the diagnosis of RLS [6]. There is an alternative RLS questionnaire, developed at Cambridge and Johns Hopkins, which may be more useful with a sensitivity of 87.2% and specificity of 94.4% [7].

## 3. RLS Prevalence

In the general population, the prevalence of RLS varies between 5–15%, and there is a clear female predominance with up to a 2 : 1 female ratio [8, 9]. Prevalence is substantially higher in certain populations such as Icelandic women where research shows RLS prevalence of 24.4% [10]. In two large studies, women were clearly found to have twice the likelihood to meet IRLSSG RLS criteria than men [11, 12]. In addition, the risk of RLS in women appears to be related to parity with an increased risk of RLS directly proportional to the number of children [8]. Of note, research from Asia shows lower prevalence rates for RLS, perhaps as low as 1.6% [13]. Overall, RLS prevalence rates appear to be higher in Caucasian populations, especially in multiparous women.

## 4. RLS Classification

RLS is classified into primary RLS and secondary RLS. Primary RLS is of an unknown etiology and seems to be related to abnormalities in the dopaminergic pathways of the central nervous system. Primary RLS appears to have a genetic component and has been described in family studies in Iceland, Quebec, and other populations. Secondary RLS is clinically diagnosed when there is a known causative process such as iron-deficiency anemia, pregnancy, end-stage renal disease, peripheral neuropathy, and multiple sclerosis [14]. 

## 5. RLS Pathogenesis

Even in the present day, the scientific understanding of RLS is poorly elucidated. There is a clear linkage to the dopaminergic pathways and iron metabolism in the substantia nigra, but the overall pathophysiology of RLS remains unclear. An autopsy-based study indicated that the etiology of RLS may be related to a defect in the regulation of transferrin receptors in the brain [15]. Radiographic imaging studies also have shown alterations in the dopaminergic receptors of the brain [16]. More recently, it has been shown that low iron levels can increase extracellular dopamine and decrease D2 dopaminergic receptors in the brain [17]. Finally, there have been genetic markers found associated with a susceptibility to PLMS [18]. Thus, while the exact mechanism of RLS pathophysiology is not fully understood, there is a clear association with iron deficiency-related dopaminergic abnormalities in the brain.

## 6. Sleep and Rheumatic Diseases

A high prevalence of sleep disturbance has been reported in rheumatic disease patients. Specifically, abnormal sleep has been reported in rheumatoid arthritis, osteoarthritis, scleroderma, Sjogren's syndrome, systemic lupus erythematosus, fibromyalgia, and spondyloarthropathy [19]. Furthermore, poor restorative sleep has been linked in this rheumatic disease population to pain, mood, fatigability, stress, and disease activity [20]. Various primary sleep disorders have been observed in RA including higher prevalence obstructive sleep apnea [21], insomnia/sleep disruption [22], and RLS [23].

## 7. RLS and Rheumatoid Arthritis

While RLS may be in part considered neurologic disease, there is a significant interplay with the overall aspect of sleep and the immune system. Besides, the issue of iron deficiency often seen, rheumatoid arthritis is a chronic inflammatory condition with numerous proinflammatory cytokines and other immunomodulatory changes present. This likely explains the association of increased rates of primary sleep disorders seen in rheumatoid arthritis, including RLS [23]. In the RA patient population, numerous investigators have reported an elevated prevalence of RLS. Almost 3 decades ago, Reynolds et al. evaluated hospitalized RA patients finding a 30% prevalence of RLS in RA compared with a control osteoarthritis group [24]. More recently, other researchers have found in RA similar prevalence rates for RLS being 27.7–31% [23, 25]. Of interest, elevated prevalence rates for RLS in other connective tissue diseases such as Sjogren's syndrome, scleroderma, and lupus appear [26]. Thus, compared to the general population, there are significantly higher prevalence rates of RLS in patients with rheumatic diseases, especially rheumatoid arthritis. It should be noted that prevalence data may be affected by subjective patient symptoms and report. Nevertheless, in recent evaluation of the prevalence of RLS in RA patients, 90.8% of symptomatic patients felt that they could differentiate their leg symptoms from their arthritic symptoms [23].

## 8. Physiology of Sleep in RA Patients

Most people sleep nearly 8 hours per day thus spending approximately one-third of their lives asleep. Sleep itself is generally divided into two main components of rapid eye movement (REM) and nonrapid eye movement (NREM) sleep. NREM sleep is further divided into stage one (S1/N1), stage two (S2/N2), and slow-wave sleep (S3 + S4/N3). During the night, individuals cycle between the various stages of sleep. During the night, there are fluctuations in hormone and cytokine levels. The hypothalamic-pituitary axis contributes to sleep regulation as does the dopaminergic system. There has been increased understanding of the importance of cytokines interacting in sleep physiology through signal regulation. It has been previously reported that IL-4, IL10, IL13, and TGF-*β* can interact to inhibit NREM sleep [27]. Other researchers have noted the bidirectional communication between the brain and immune system which has major implications for sleep physiology especially if compromised by a proinflammatory condition such as RA [28]. An area of concern involves the relationship showing that some inflammatory mediators (IL-1*β* and TNF-*α*) have a diurnal rhythm with elevated levels during sleep [29]. This is of interest given the use of immunomodulating medications for RA. Since RA can be associated with increased TNF-*α*, it is intriguing what effect biologic therapies may have on sleep [30]. In one recent study, researchers found an improvement in sleep physiology for RA patients treated with infliximab [31]. In another PSG-based study of RA patients, there was improved sleep efficiency after anti-TNF-*α* therapy [32]. A large (but not PSG-based) RA population study failed to show a significant difference in subjective sleep scores of patients treated with anti-TNF medications [33]. However, more recent trials assessing subjective sleep outcomes show benefit of immunomodulatory interventions (MTX, adalimumab, abatacept) in RA patients [34, 35]. Thus, there is evidence of a bidirectional process in sleep physiology and the immune systems with potential for poor sleep to affect RA and RA to affect sleep. Finally, a recent study found interesting differences in hepcidin levels in RA patients with anemia of chronic disease versus iron deficiency anemia [36]. Since IL-6 has been linked both to the pathogenesis of RA as well as the production of hepcidin, it may be possible for an anti-IL-6 receptor antibody to improve RA symptoms, reduce hepcidin levels, and improve RLS symptoms.

## 9. Treatment for RLS

Since the main pathophysiologic abnormality of RLS is due to alterations in the dopaminergic pathways, treatment options are focused around both nonspecific medications and then specific dopamine receptor agonists. The initial diagnostic workup of RLS should include evaluation of iron stores including serum ferritin levels, TIBC. In a recent randomized, blinded study, general population patients who met the 2003 IRLSSG criteria for RLS and had low serum ferritin were treated with iron versus placebo and were found to have clinically significant improvement in their RLS symptoms/severity as well as a trend towards improvement in quality of life [37]. Other studies have shown variable benefit of iron replacement therapy in RLS patients [38]. There are no studies to date that have proven this benefit in the RA RLS population. Nevertheless, in a patient with rheumatoid arthritis, RLS symptoms meeting the IRLSSG criteria, and low serum ferritin levels, it would be advisable to start with iron replacement therapy. Assuming a normal ferritin level (or lack of improvement with iron replacement), the next step in RLS treatment is a dopamine-specific agent. At present, there are three newer synthetic dopamine agonists that are FDA approved for RLS treatment in the USA. These medications include ropinirole, pramipexole, and rotigotine. In addition, sustained-release gabapentin enacarbil has also been FDA approved for RLS therapy. While these first-line therapies are quite effective in managing RLS symptoms and severity, there are potential issues. These adverse effects include the potential for compulsive behavior disorders associated in up to one-third of patients with dopamine agonists at target dosing levels [39]. Other side effects associated with dopamine agonist therapy include the development of valvular heart disease and also a higher incidence of congestive heart failure (appears limited to pramipexole) [40]. Thus, while these are recommended first-line treatments for RLS, patients should be first appropriately diagnosed and a physician experienced in RLS therapy should discuss potential risks/benefits with the patient.

## 10. Summary

Chronic fatigue and unrefreshing sleep are common complaints in patients with rheumatic diseases such as rheumatoid arthritis. While a number of sleep abnormalities have been reported in RA, RLS is a frequent comorbidity with prevalence rates of up to 30% of all RA patients. Most of these patients are able to distinguish the RLS symptoms from their RA symptoms. Sleep and TNF-*α* levels appear to have a linkage, and anti-TNF therapy may potentially improve sleep both by improving RA-related joint pain and also by a cytokine-mediated process. RLS is an underdiagnosed condition which can seriously impact on sleep and overall quality of life for these individuals. Since there are current treatments that can significantly improve RLS symptoms/severity, it is important to screen rheumatic disease patients for sleep abnormalities. 
